# Urine metabolomics based prediction model approach for radiation exposure

**DOI:** 10.1038/s41598-020-72426-4

**Published:** 2020-09-30

**Authors:** Ritu Tyagi, Kiran Maan, Subash Khushu, Poonam Rana

**Affiliations:** 1 Metabolomics Research Facility, Institute of Nuclear Medicine and Allied Sciences (INMAS), DRDO, S. K Mazumdar Road, Timarpur, Delhi, 110054 India; 2 NMR Research Centre, Institute of Nuclear Medicine and Allied Sciences (INMAS), DRDO, Delhi, India

**Keywords:** Metabolomics, Predictive markers

## Abstract

The radiological incidents and terrorism have demanded the need for the development of rapid, precise, and non-invasive technique for detection and quantification of exposed dose of radiation. Though radiation induced metabolic markers have been thoroughly investigated, but reproducibility still needs to be elucidated. The present study aims at assessing the reliability and reproducibility of markers using nuclear magnetic resonance (NMR) spectroscopy and further deriving a logistic regression model based on these markers. C57BL/6 male mice (8–10 weeks) whole body γ-irradiated and sham irradiated controls were used. Urine samples collected at 24 h post dose were investigated using high resolution NMR spectroscopy and the datasets were analyzed using multivariate analysis. Fifteen distinguishable metabolites and 3 metabolic pathways (TCA cycle, taurine and hypotaurine metabolism, primary bile acid biosynthesis) were found to be amended. ROC curve and logistic regression was used to establish a diagnostic model as Logit (*p*) = log (*p*/1 − *p*) = −0.498 + 13.771 (tau) − 3.412 (citrate) − 34.461 (α-KG) + 515.183 (fumarate) with a sensitivity and specificity of 1.00 and 0.964 respectively. The findings demonstrate the proof of concept and the potential of NMR based metabolomics to establish a prediction model that can be implemented as a promising mass screening tool during triage.

## Introduction

The increasing burden of natural background radiation and terrestrial radionuclides is a major concern for exposure to radiation to the mass population. According to U.S. commission report on prevention of munitions for terrorism and mass destruction^1^, accidental radiation exposure is a matter of global worry that needs emergency preparedness, policy makers to prevent undesirable exposures as well as to treat and manage exposed individuals. Additionally, the accidental nuclear disasters of Fukushima and the growing risks of radiological terrorism demanded the development of biomarkers of ionizing radiation and countermeasures for rapid and accurate measurement of absorbed radiation dose for mass screening of exposed individuals^2–4^. There is a clear need for high throughput biomonitoring and diagnostic platform that is easily deployable, rapid, reproducible, reliable, and appropriate to triage and permit rapid assessment of possibly massive radiation exposure casualties. Recognition of predictive biomarker(s) that have the potential to detect radiation induced syndromes at the earliest preceding the onset of organ specific damage is the need of the hour^2,5,6^.

Metabolomics is one such high-throughput technology that has great potential for radiation bio-dosimetry that can play an imperative role during the initial triage of radiological disasters to characterize the metabolic status of an individual before the onset of symptoms. It is about a decay old omics technique and in the last few years, its application has been extended towards qualitative and quantitative assessment of ionization radiation exposure induced response mainly in biological fluids, serum and urine^7–14^. Liquid chromatography–mass spectrometry (LC–MS) based studies have elucidated many urinary metabolic markers for radiation exposure pertaining to oxidative stress, energy metabolism, DNA damage, inflammation, and tissue damage^4,15,16^. NMR spectroscopy based metabolomics due to its ease and reproducibility over LC–MS has allowed a time and dose responsive comprehensive coverage of urinary metabolites in toxicological and clinical research. However, NMR based metabolomics has been less explored in the case of radiation research. Metabolomics reveals the pathophysiological perturbations to metabolic pathways that could serve as predictive markers for possible health outcomes and therefore, may serve as targets for therapeutic intervention post radiation exposure. Performance evaluation and final predictive model testing of biomarkers are absolutely required for translational research or for the development of a high throughput bio-dosimetry device for mass screening. Primarily, role of metabolomics in the development of biomarker is to focus on building a predictive model that could be used to classify unidentified sample(s) into specific groups with the best specificity and sensitivity^17^.

Our previous published study has demonstrated radiation induced patho-physiological perturbations in urine using ^1^H NMR based metabolomics wherein, metabolite markers were found to be associated with energy metabolism, amino acids, and gut flora metabolism^18^. However, reproducibility, validation, and estimation of sensitivity and specificity of identified markers are the prerequisite step towards the translational approach. Therefore, the present study has been conceived to provide a proof of concept and establish a model for identification of radiation exposure based on metabolite markers identified through NMR spectroscopy. To the best of our knowledge, this is the first study that looks into the potentiality of the prediction model approach for the identification of NMR spectroscopy based radiation markers using a logistic regression model.

## Results

### Metabolomic analysis of urine

**Figure 1:**
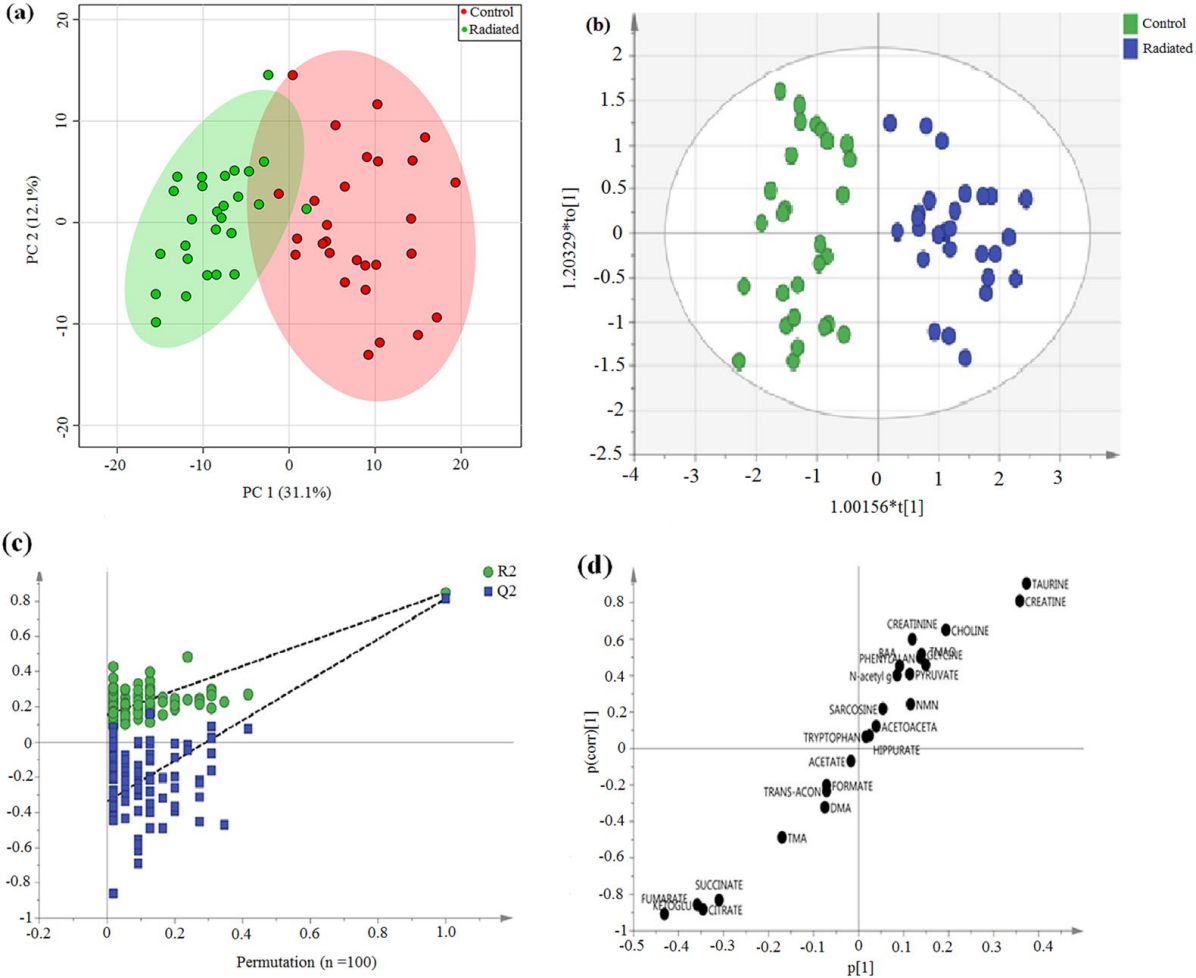
Multivariate analysis derived from ^1^H NMR spectra of urine sample from control and irradiated animals. (**a**) PCA score plot, (**b**) OPLS- DA score plot, (**c**) OPLS-DA validation plot (permutation times n = 100) and (**d**) The OPLS-DA derived corresponding S-plot.

Representative ^1^H NMR spectra with identified metabolites are shown in supplementary Fig. 1. The competency of ^1^H NMR based metabolomics approach to differentiate irradiated from non-radiated controls was assessed using multivariate analysis in our study. Principal component analysis (PCA) score plot displayed clear separation between the irradiated and control group (Fig. 1a). From the corresponding loading plots of PCA, the chemical shifts values that lied far away from origin has the greatest contribution and substantially responsible for demarcation between irradiated and control group and therefore may be regarded as the discriminating metabolites for the irradiated group. Based on PCA, 24 metabolites were identified (supplementary Fig. 2), which were then used to perform supervised clustering, orthogonal partial least squares discriminant analysis (OPLS-DA) (Fig. 1b). The analysis produced a strong model with high validated predictability (Q^2^ cum = 0.813) and goodness of fit value (R^2^ cum = 0.85). The values greater than 0.5 indicates that model possessed a reasonable fit with good predictive power^19,20^. The permutation plot with n = 100 permutations further confirmed the constructed OPLS-DA model was positive and valid as all permutated values were lower than the original values at the right and the line plot intercepts Y axis below zero (Fig. 1c). The corresponding S-plot shows the potential 6 metabolites responsible for demarcation between the control and irradiated group (Fig. 1d). Overfitting of the OPLS-DA model was further confirmed by cross validated ANOVA (CV ANOVA) and highly significant *p* value (1 × e^−17^) of CV ANOVA score suggests the strengthening and robustness of the model since score can be used as a guide for the optimal fitting of a model^20^.

### Identification of important differential metabolites

**Figure 2:**
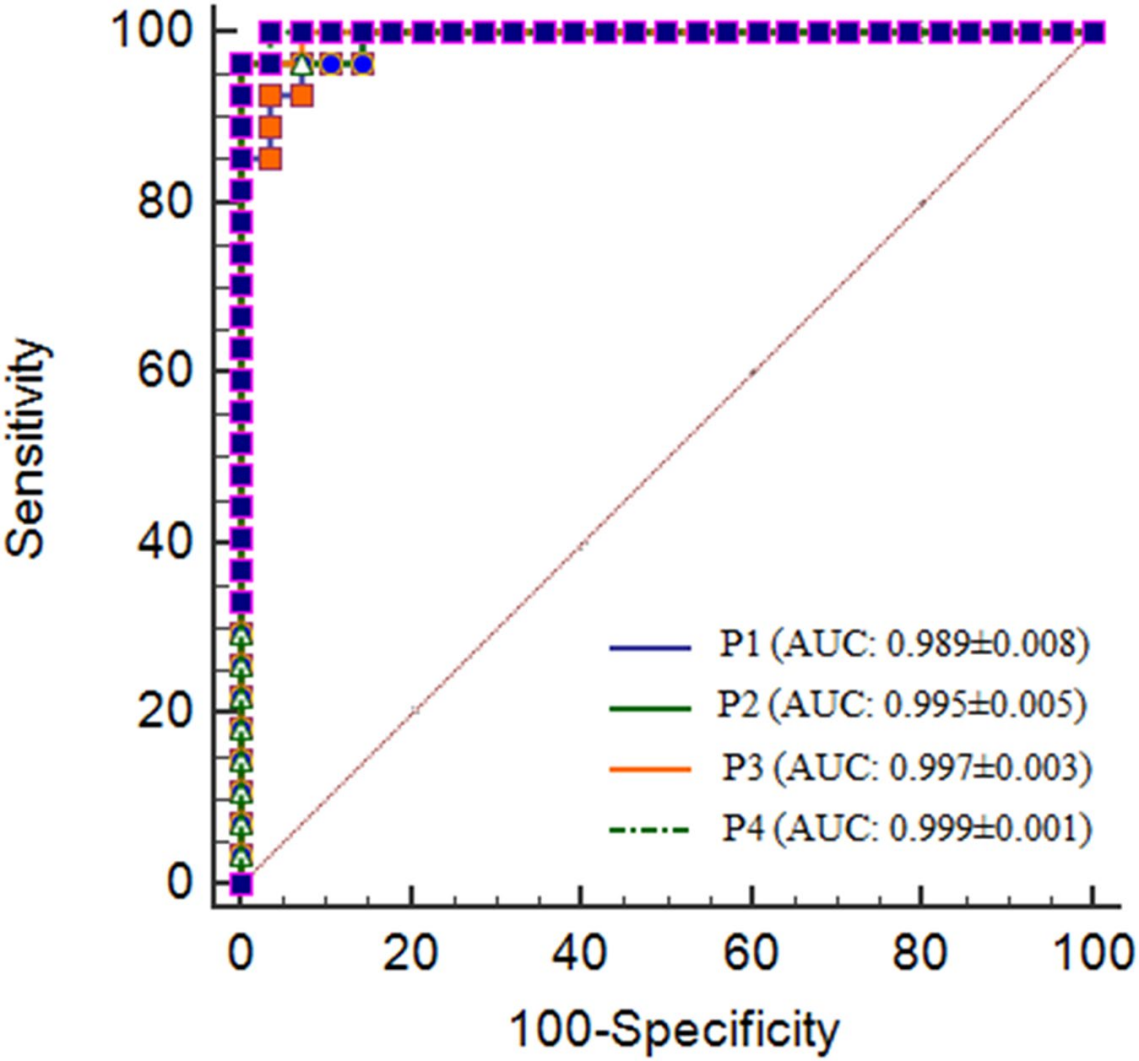
The ROC analysis results from the four prediction models calculated from the logistic regression analysis. The diagnostic performance of each biomarker model was assessed by the area under the ROC curve (AUC) and the determination of sensitivity and specificity at the optimal cut-off was determined using the Youden index (J). The optimized model was the P4 model with an AUC of 0.999 (95% CI 0.933–1.00).

PCA analysis identified 24 metabolites, however, based on the correlation coefficient with threshold value of (∣r∣ > 0.3) (r > 0.3 and r < −0.3), out of the 24 metabolites, 15 differential metabolites were identified (Table 1). Further, supervised analysis was able to detect 6 metabolites out of 15 metabolites on the basis of VIP score (> 1) and visualized S plot. These 6 differential metabolites i.e. taurine, creatine, succinate, citrate, α- ketoglutarate (α-KG) and fumarate were then used to perform predictive modeling using stepwise logistic regression analysis. The result ascertained that among these 6 metabolites, only 4 metabolites i.e. taurine, citrate, α- ketoglutarate (α-KG) and fumarate had the greatest and significant contribution to the combined model between irradiated and control groups (Table 2a) as the model using all 6 differential metabolites could not be converged. Therefore the final model was built using 4 metabolites only. The diagnostic performance of the model and individual metabolites was quantified through the Receiver operating characteristic (ROC) curve. All six metabolites had an area under the curve (AUC) > 0.90 depicting high range of sensitivity (77.78—100%) and specificity (89.29—100%) (Table 2b and supplementary Fig. 3). However, the combined metabolites were the better discriminator than each metabolite individually as depicted in Table 2a and supplementary Fig. 3 indicating strong diagnostic performance of combined biomarker panel. The final diagnostic model was expressed as: Logit (*p*) = log (*p*/1 − *p*) = − 0.498 + 13.771 (tau) − 3.412 (citrate) − 34.461 (α-KG) + 515.183 (fumarate). The corresponding ROC curve had an AUC of 0.999 (95% confidence interval (CI) 0.933 to 1.00) with a Youden index J of 0.964 and sensitivity and specificity of 100 and 96.4 respectively (Fig. 2). The training/discovery set displayed a sensitivity of 0.947 and specificity of 1.00 with an AUC of 0.998. The model was also validated with tenfold cross validation which had a sensitivity, specificity, and AUC of 0.926, 0.893, and 0.889 respectively (supplementary Fig. 4). This further verifies the robustness of the diagnostic model for the distinctive classification of irradiated and control groups.

**Table 1:** List of 15 Key metabolites responsible for discriminating irradiated and control group.

Metabolites	HMDB ID	*p* value^a^	VIP^b^	R^c^	Fold change^d^
Taurine	HMDB0000251	3.49 × 10^−^15	1.70	0.85	0.31
Citrate	HMDB0000094	1.74 × 10^−^13	1.58	− 0.83	2.54
Creatine	HMDB0000064	5.08 × 10^−^12	1.69	0.77	0.29
αKG	HMDB0000208	1.63 × 10^−^13	1.66	− 0.75	3.49
Fumarate	HMDB0000134	6.37 × 10^−^15	2.00	− 0.70	4.69
Succinate	HMDB0000254	4.62 × 10^−^12	1.47	− 0.69	2.40
Choline	HMDB0000097	1.84 × 10^−^08	0.86	0.69	0.62
Creatinine	HMDB0000562	6.13 × 10^−^05	0.55	0.54	0.84
Glycine	HMDB0000123	1.41 × 10^−^04	0.66	0.52	0.71
Phenylalanine	HMDB0000159	1.35 × 10^−^03	0.65	0.48	0.75
Pyruvate	HMDB0000243	5.22 × 10^−^03	0.74	0.45	0.80
TMAO	HMDB0000925	1.42 × 10^−^03	0.75	0.42	0.71
Branched amino acids (BAA)	HMDB0000687 (l-Leucine), HMDB0000172 (l-Isoleucine), HMDB0000883 (l-Valine)	2.10 × 10^−^03	0.49	0.40	0.86
TMA	HMDB0000906	2.15 × 10^−^03	0.82	− 0.40	1.53
N-Acetyl glycoprotein	HMDB0000215	1.38 × 10^−^02	0.40	0.40	0.88

^a^*p* values were derived from two-tailed Student’s *t* test.

^b^Variable Importance in the projection (VIP) was obtained from OPLS DA with a threshold of 1.0

^c^Correlation coefficient was obtained from OPLS DA with a threshold of 1.0

^d^Positive values indicate higher levels in irradiated group and negative values indicate lower levels in irradiated group.

**Table 2:** Prediction models from the logistic regression and the ROC analysis results of the combined (a) and individual metabolites (b).

	Prediction models	AUC	SE^a^	95% CI^b^	Youden index (J)	Sensitivity	Specificity
**(a)**							
P1	*p* = [1/1 + e^−^{−9.026 + 4.319 * taurine}]	0.989	0.0084	0.916–1.00	0.891	96.3	92.86
P2	*p* = [1/1 + e^−^{−4.161 + 3.697 * taurine−0.894 * citrate}]	0.995	0.0058	0.925–1.00	0.963	96.3	100.00
P3	*p* = [1/1 + e^−^{−5.057 + 6.006 * taurine-0.500 * citrate-5.335 * α keto glutarate}]	0.997	0.0032	0.930–1.00	0.963	96.3	100.00
P4	*p* = [1/1 + e^−^{−0.498 + 13.771 * taurine-3.412 * citrate-34.461 * α keto glutarate + 515.183 * fumarate}]	0.999	0.0018	0.933–1.00	0.964	100.00	96.4
**(b)**							

	Metabolites	AUC	SE^a^	95% CI^b^	Youden index (J)	Sensitivity	Specificity
1	Citrate	0.966	0.033	0.878–0.996	0.927	96.3	96.43
2	Taurine	0.989	0.0084	0.916–1.00	0.891	96.3	92.86
3	Fumarate	0.976	0.0192	0.893–0.999	0.927	96.3	96.43
4	α-Ketoglutarate	0.955	0.0298	0.862–0.993	0.892	100.00	89.29
5	Creatine	0.954	0.0238	0.861–0.992	0.777	77.8	100.00
6	Succinate	0.954	0.0256	0.861–0.992	0.817	88.89	92.86

^a^Standard error.

^b^Confidence interval.

### Identification of metabolic pathways

**Figure 3:**
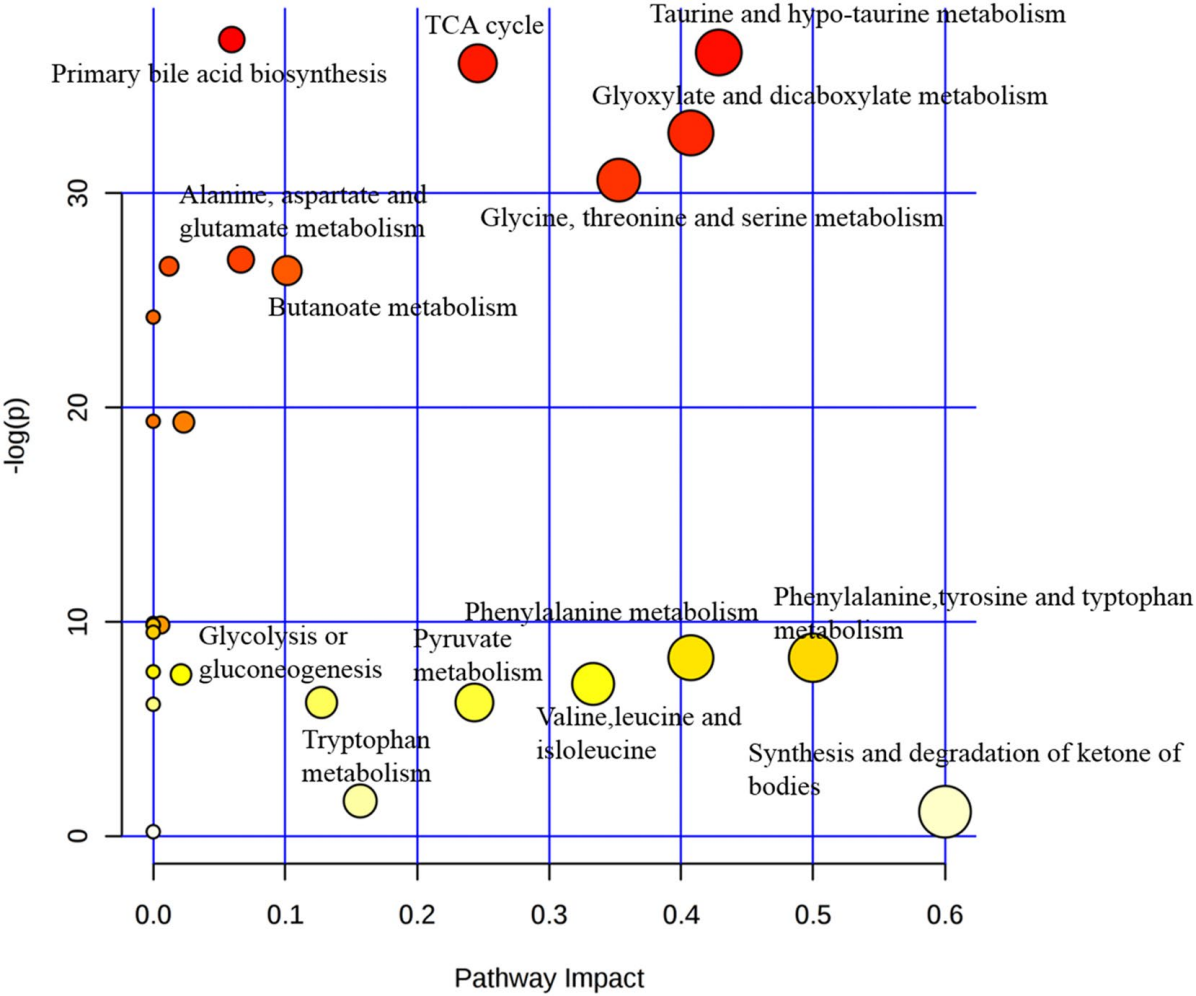
Metabolic pathway mapping of the impacted metabolic network identified between control and irradiated group. The χ-axis represents the pathway impact, and the y-axis represents the − log (*p*).

To recognize the metabolic pathways that were disturbed due to radiation, metabolic pathway analysis was carried out on 15 most contributory metabolites with |r| > 0.3 and *p* < 0.05. Out of the 28 pathways analyzed, 3 pathways with pathway impact > 0.05, −log (*p*) > 35 and with good hits (5, 1 and 3 for Tri-carboxylic acid (TCA) cycle, taurine and hypotaurine metabolism and primary bile acid biosynthesis respectively) were considered. Of all the 3 pathways analyzed the taurine and hypotaurine pathway was recognized as the most considerably altered pathway between control and irradiated group with highest impact value of 0.43. For the TCA cycle among the total of 20 metabolites present in the pathway, 5 metabolites (succinate, pyruvate, α-K.G., fumarate and citrate) has been found altered in the present study (Fig. 3).

## Discussion

Nuclear accidents, dispersal of radiological devices and exposure of radioactive materials to mass population or individual demanded the need to develop fast and trustworthy means of biological dosimetry for recognition of radiation exposed individuals. Though cytogenetics is the gold standard for radiation bio-dosimetry however, metabolomics also has the potential for the first level of assessment of exposed individuals. It is nearly impossible to get the human subjects/patients exposed to whole body radiation exposure, as therapeutic radiation cannot mimic whole body radiation exposure scenarios of any radiological incident or accident. Also, the patients undergoing radiation therapy either receive localized or focal radiation and constitute a heterogeneous population based on the dose and area of radiation. Moreover, because of comorbidity, these patients cannot become true models/subjects of radiation exposure. Therefore, the present study has been designed in preclinical model to provide a proof of concept to an established prediction model for whole body radiation exposure using NMR based metabolomics. Urinary metabolomics has evolved as one of the most promising biomarker platform as urine can be easily collected and is more stable compared to other bio-fluids. Urinary radiation metabolomics has identified radiation induced metabolic markers however, the reproducibility, reliability, and validation of these markers still need to be elucidated. The determination of sensitivity and specificity of these metabolic markers is very much essential for delineation of early exposure to radiation and thereafter early/prompt intervention.

For disease model, metabolomics have been widely utilized for single biomarker discovery however, multiple biomarkers or panels might be better for discovering candidates with higher specificity and sensitivity. Thus the aim of this study is to assess the utility of multiple biomarkers for radiation exposure using multivariate analysis and logistic regression using stepwise variable selection method.

PCA revealed clear differences between controls and irradiated groups. However, OPLS DA may be an appropriate diagnostic model as it is a better discriminator in categorizing irradiated and control groups. The established prediction model based on four markers, i.e. taurine, citrate, α-KG and fumarate with high sensitivity, specificity, and AUC suggests that they could be the candidate biomarkers for radiation exposure. It also indicates that individual metabolic biomarkers cannot be ideal for radiation exposure screening. The pathway analysis has shown that these metabolites are closely linked with the citric acid cycle, hypotaurine and taurine metabolism and primary bile acid biosynthesis.

With the use of multivariate statistical analysis and prediction modeling, the findings of our earlier study have been validated and show reproducible results. In the last few years, many LC–MS metabolomics studies have identified many metabolites associated with radiation exposure^16,21^. However, there are few NMR based studies that have exploited the potential of metabolomics for the identification of potent metabolite markers^22–25^. Undoubtedly the sensitivity of LC–MS is way ahead of NMR for profiling but the ease of acquisition, identification of metabolites and reproducibility of information in spectrum makes NMR based metabolomics a suitable and comfortable platform in biomarker identification. Notably, the present, as well as earlier study, have shown several NMR based metabolite markers associated with energy metabolism (citrate, α- K.G, succinate, fumarate), amino acids (leucine/Isoleucine), creatine, creatinine, hippurate and taurine overlapping with LC–MS studies^6,18,25–27^. Few of the identified metabolite markers in the present study are also in accordance with other radiation metabolomics studies^6^. The present study and the recent literature suggest taurine and citrate amongst the candidate biomarkers for radiation exposure. Therefore, ease and reproducibility of information obtained by NMR spectroscopy make these markers as candidate markers for radiation exposure.

Taurine and hypotaurine metabolism has the highest impact score in our metabolic pathway analysis. Among the known urinary metabolites of radiation exposure taurine is well documented, but its exact cause for its upregulation following radiation is still not known. Its biological function includes bile acid conjugation and it also acts as anti-oxidant to protect the body by inhibiting reactive oxygen species (ROS)^28^. It might play a direct protective role for cell by preventing the ionic and water shifts that lead to cellular damage and death. The increased urinary excretion of taurine post high dose of ionizing radiation might be due to cellular damage (i.e. damage to the plasma membrane) that results in enhanced passive leakage of taurine out of cells^29^. Additionally, tissue injury induced by radiation results in a greater concentration of circulating sulfur-containing amino acids that are excreted in the urine in terms of excess taurine^29^. Another proposed mechanism includes the destruction of circulating lymphocytes post radiation exposure^30^.

The TCA cycle identified in our pathway analysis provides evidence of mitochondrial dysfunction post irradiation. The radiation induces oxidative stress slows down the mitochondrial TCA cycle to minimize the generation of free radicals^31^. The reduced excretion of energy metabolites (citrate, α-KG, succinate, and fumarate) further suggest perturbed energy metabolism and are indicative of the radiation induced oxidative stress.

The metabolites creatinine and creatine were also found to be increased post radiation exposure. One of the previous study has reported creatine as a biomarker for 6.5 Gy dose at 24 h post irradiation^16^. Additionally, creatine is also a biomarker of radiation exposure in non-human primates (*Macaca mulatta*)^32^. Creatinine is formed from creatine and creatine phosphate and increased excretion of both (creatinine and creatine) indicates incapability of irradiated muscle to use creatine^33^.

To best of our knowledge, this is the first study to recognize a set of NMR spectroscopy based biomarkers that can differentiate between control and irradiated group with such high sensitivity, specificity and accuracy based on logistic regression based prediction modeling in preclinical setup. In the present study, using a prediction model approach a proof of concept has been provided for whole body radiation exposure, however, this work will be further extended with a range of radiation doses.

## Conclusion

The present findings of our work have provided a strong foundation for the potential application of NMR based metabolomics approach for screening of triage during nuclear accidental scenario. However, limiting the human subjects with radiation exposure mimicking or close to real nuclear accident, the findings need to be scaled up to humans using advanced mathematical modeling and that needs extensive data set generated from a range of radiation dose exposure and would be a straight away expansion of the current study.

## Materials and methods

### Chemicals

All chemicals, trimethylsilyl-2,2,3,3-tetradeuteropropionic acid (TSP), NMR solvents, and deuterium oxide (D_2_O) used during the study were obtained from Sigma-Aldrich (St. Louis, MO, USA).

### Animal handling and radiation exposure

Sixty ‘C57BL/6’ male mice, 8–10 weeks of age and 25–35 g of body weight obtained from the animal facility of the institute were used for this study. Out of the 60 mice, sufficient urine was obtained only from 55 mice. Prior to group allocation and whole body radiation exposure, the animals were acclimatized in polypropylene cages for 48 h under standard room temperature of 19–23 °C, humidity in range of 45–65% and fluorescent lighting was provided for 12 h light/12 h dark cycle. Food and water were provided ad libitum. The animals after acclimatization were randomly allocated into two groups. One group with (n = 27) animals were exposed to 7.5 Gy whole body radiation dose through the Tele ^60^Co gamma irradiation facility at our institute (Bhabhatron II, Panacea, India) with source operating at 1.568 Gy/min. Mice were exposed to 7.5 Gy of whole body radiation with a field of view of 30 × 30 cm^2^ and surface to source distance (SSD) of 80 cm. A whole body radiation dose of 7.5 Gy in mice is approximately equivalent to 3.375 Gy of human and is considered to be associated with a moderate dose of radiation^26,34,35^. Rest of the animals (n = 28) were sham irradiated and served as a control group. None of the animals were anaesthetized during sham or radiation exposure. All experimental protocols and animal handling were approved and according to the strict guidelines of the institutional animal ethical committee at the institute of nuclear medicine and allied sciences (INMAS), DRDO, Delhi-110054 (8/GO/RBI/S/99/CPCSEA/INM/IAEC/2017/09).

### Sample collection

Prior to urine collection, all the animals were kept in clean metabolic cages for 3 days for acclimatization. Urine samples were collected in ice-cooled tubes containing 1% sodium azide (n = 27) at 24 h for post irradiation group and (n = 28) for the control group. The criteria for choosing this time point was based on our previous findings that have shown maximum metabolic perturbations at 24 h after 8 Gy of WB radiation and also in line with the literature^6,27^. Urine samples were centrifuged and the supernatant of urine so obtained was stored at − 80°C for further NMR spectroscopic analysis.

### NMR analysis

Three hundred fifty microliter of thawed and centrifuged urine was mixed with 250 µl of 0.2 M deuterated phosphate buffer (pH 7.4) with 1 mM TSP and transferred to 5 mm NMR tubes. ^1^H NMR spectra of urine samples were recorded at 298 K on a narrow bore NMR spectrometer operating at 700 MHz (Agilent, USA). 1D spectra was acquired with NOESY using a water presaturation pulse. The parameters used for the 1D experiment were as follows: dummy scan = 4, relaxation delay of 5 s, mixing time of 50 ms and acquisition time of 25 s was used. Sixty four FIDs were collected into 32 K data points over a spectral width of 6,410.25 Hz. The FID was weighted by an exponential function with a 0.3 Hz line broadening factor prior to Fourier transformation. The data was pre-processed using topspin software. Individual metabolites were identified using published literature using Human Metabolome Database (HMDB).

### Multivariate statistical data analysis

The statistical expression of different metabolites in control and radiated group were analyzed using multivariate statistical data analysis. The multivariate analysis was performed in a total of 55 spectra of which 27 are from the radiated group and n = 28 from the control group. All spectra were phased and corrected manually for baseline using TOPSPIN 2.1 (Bruker, Germany). The corrected NMR spectra with spectral range of δ 0.5–9.5 were imported into AMIX (Bruker, Biospin, Germany) and all the spectra were segmented into 901 bined region of equal width of 0.01 ppm. Prior to any statistical analysis, regions for water (4.5–5.0 ppm) and urea (5–6.0 ppm) were eliminated and the data were pre-processed using scaling and normalization. The pre-processing is done to eliminate the possible bias that could arise either due to sample variability or handling or both. Normalization (by sum) was performed to minimize possible differences in concentration between samples. Following normalization, pareto-scaling (mean-centering and division by the square root of the standard deviation of each variable) was done to give all variables equal weight regardless of their absolute value. After data pre-processing, an unsupervised pattern recognition (PR) method PCA was done to detect systemic variation and intrinsic clusters within the data set.

Based on PCA, the spectral region (bins) differentiating between controls and radiated group were identified, integrated and the relative intensity of each of the identified metabolites was calculated. Relative intensities were then further used to perform the univariate and supervised multivariate analysis. A supervised PR method OPLS-DA was done to achieve maximum separation of control and irradiated group. OPLS-DA model was validated using CV ANOVA which provides *p* value that indicates the level of significance for group separation in OPLS analysis. A permutation test was used to validate the predictive capability of the computed OPLS-DA model. The most relevant potential markers were identified using the S plot that was constructed from the loading plots of OPLS-DA. Additionally, univariate analysis such as for fold change and correlation analysis was also carried out for the identified metabolites. To facilitate the interpretation of the findings, correlation coefficient of both groups were then calculated. In the present study, a cut-off value of |r| > 0.3 (r > 0.3 and r < − 0.3) was chosen for correlation coefficient as significant based on the discrimination significance (*p* < 0.05). Finally, based on correlation coefficient, VIP and S plot, the specific metabolite responsible for differentiation of control and radiated group were identified.

### Selection of potential biomarker candidates

The selected metabolites were then used to construct the prediction model and the accuracy of the model was evaluated using the ROC curve. A stepwise forward logistic regression model was constructed to design the best metabolite combination. ROC curve analysis was carried out to evaluate the combined model following the DeLong method. The importance and demonstration of metabolites as a biomarker model were assessed using sensitivity, specificity, and AUC, at the optimum cut off defined by the Youden index (maximum vertical distance).

### Multivariate power analysis calculation

The minimum number of animals required to attain statistical significance between the radiated and control groups was determined by the power analysis option of Metaboanalyst 3.0. The input data for sample size estimation consisted of 24 key metabolite variables used for OPLS-DA analysis. With an FDR-adjusted *p* value of 0.004, the sample size of 3, 10, 16, 24, 40 and 60 per group were calculated with predicted test powers of 0.89, 1.00, 1.00, 1.00 and 1.00 (supplementary Fig. 5), and this clearly verify that the sample sizes selected for this study were indeed more than sufficient.

### Metabolic pathway analysis

To appreciate the inherent and complex network properties of various metabolites, detailed analysis of the most significant metabolic pathways between irradiated and control groups was performed by Metaboanalyst 3.0. For metabolic pathway analysis, Metaboanalyst software makes use of a high-quality KEGG database. The software also uses algorithms and concepts that include Global Test and Global Ancova, as well as pathway topology analysis. The *p* value and the pathway impact value are calculated using enrichment and pathway topology analysis respectively. The most impacted metabolic pathway was set as pathway impact > 0.05 and −log (*p*) > 35.

### Statistics

Multivariate analysis of NMR data and pathway analysis were performed using SIMCA-P version 14.0 (Umetrics AB, Umea, Sweden) and Metaboanalyst 3.0 (www.metaboanalyst.ca) respectively. The ROC curve for prediction modelling was performed using MedCalc software version 19.0.7 (Broekstraat, Mariakerke, Belgium). The measurement data are expressed as the mean ± SE (standard error). For univariate analysis, the differences between the two groups were analyzed using Student’s *t* test. The level of significance was set at *p* < 0.05.

## Supplementary information

Supplementary Information.
