# Supplementary material for: Urine metabolomics based prediction model approach for radiation exposure
Source: Sci Rep. 2020 Sep 30;10:16063. doi: 10.1038/s41598-020-72426-4 (PMC7527994; doi:10.1038/s41598-020-72426-4)
Supplement: Supplementary file 1 — Supplementary Information. [file 41598_2020_72426_MOESM1_ESM.docx]

**Urine Metabolomics Based Prediction Model Approach for Radiation Exposure**

Ritu Tyagi^†^, Kiran Maan^†^, Subash Khushu^1^, Poonam Rana*

Metabolomics Research Facility, Institute of Nuclear Medicine and Allied Sciences (INMAS), DRDO, Delhi, India, ^1^NMR Research Centre, Institute of Nuclear Medicine and Allied Sciences (INMAS), DRDO, Delhi, India

^†^ Both the authors have an equal contribution.

*Corresponding Author:

Dr. Poonam Rana, Ph. D

Metabolomics Research Facility

Institute of Nuclear Medicine and Allied Sciences (INMAS), DRDO

S. K Mazumdar Road, Timarpur, Delhi-54, India

Tel: 91-11-23905319

Email address: [poonam@inmas.drdo.in](mailto:poonam@inmas.drdo.in)


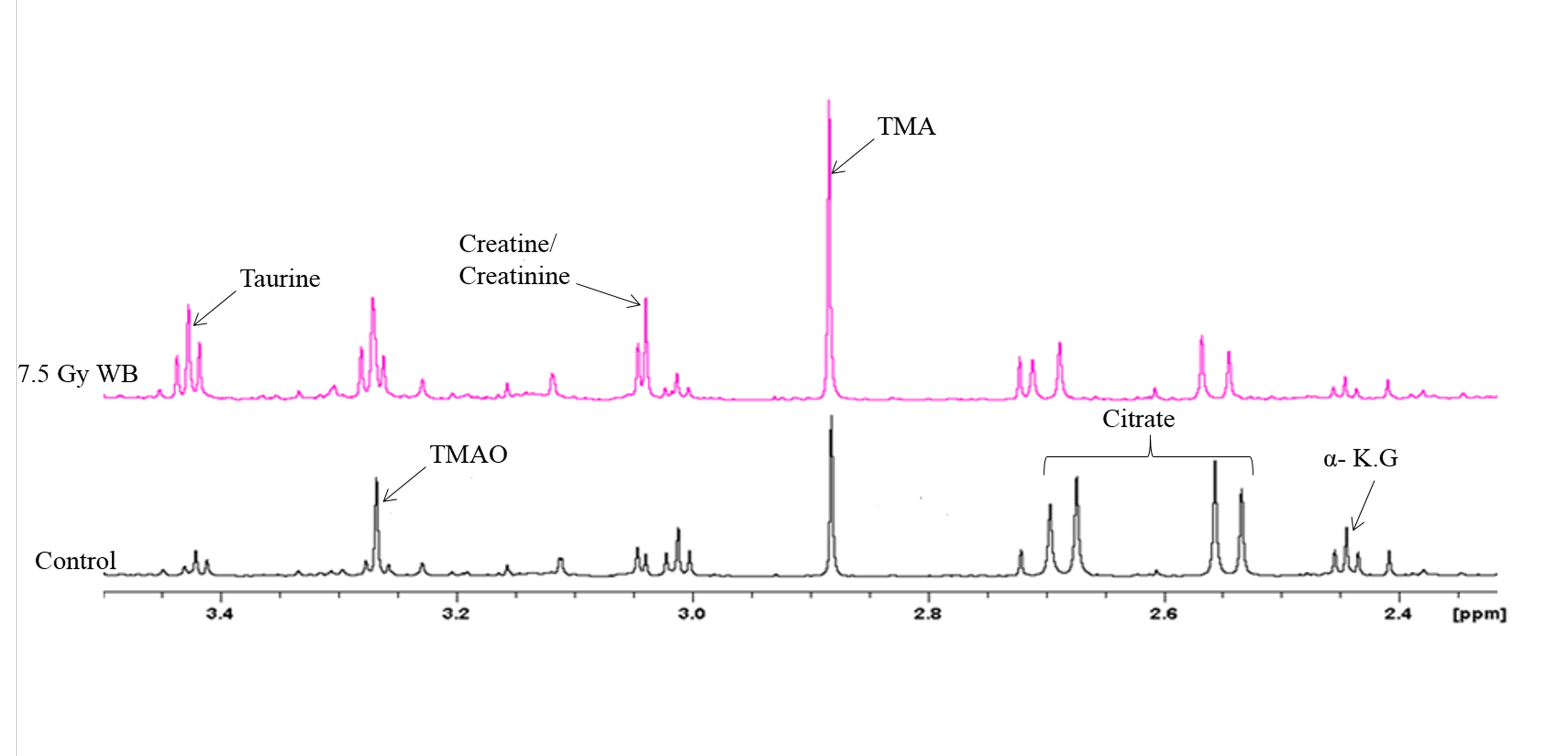


**Legend for Supplementary Figure 1:**

Representative ^1^H NMR spectra of urine from control and radiation group


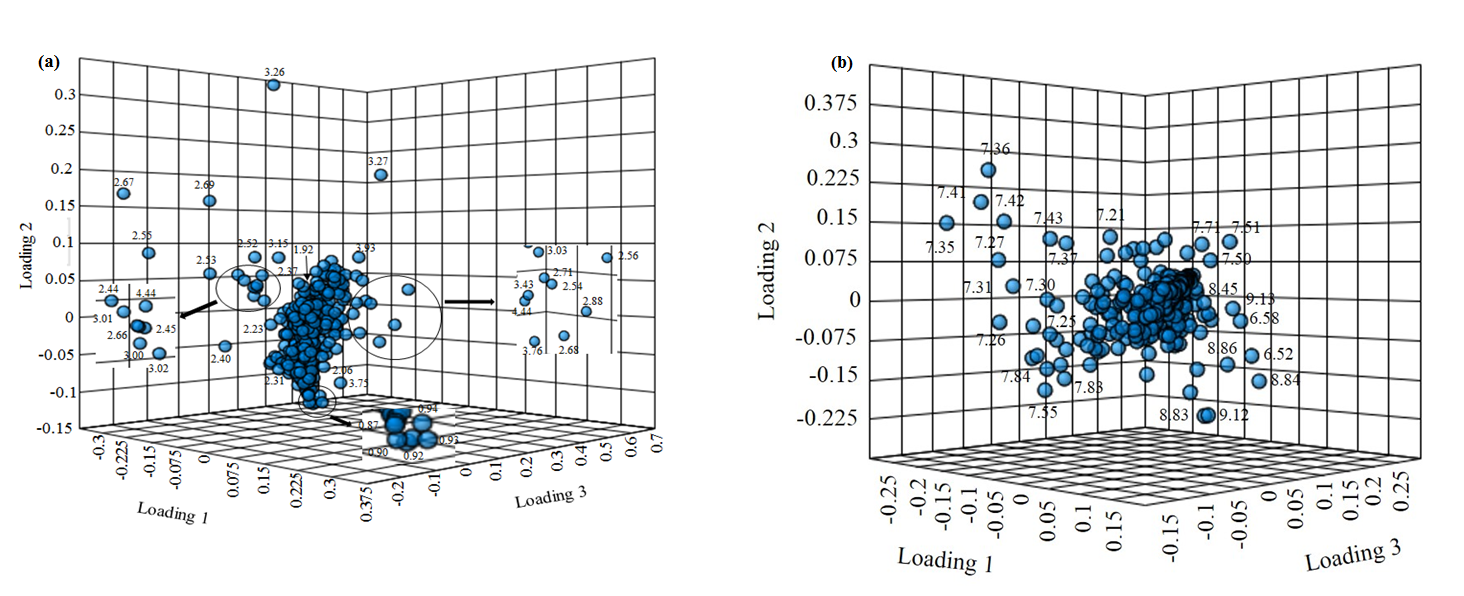


**Legend for Supplementary Figure 2:**

Representative PCA loading plot (a) 0.5-4.5 ppm and (b) 6.0-9.5 ppm based on ^1^H NMR spectra of urine sample at 24 h from control and irradiated mice


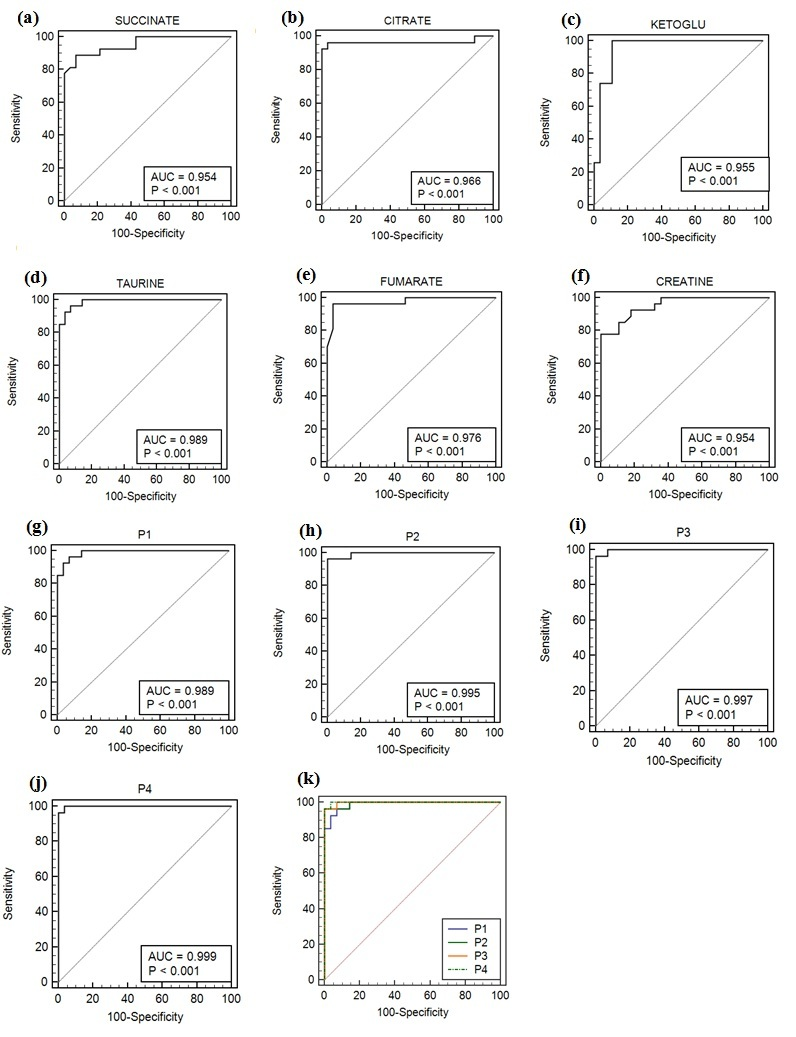


**Legend for Supplementary figure 3**

Representative ROC curve analysis based on logistic regression analysis for individual metabolite (a-f), all four individual (g-j) and combined (k) predictive model.


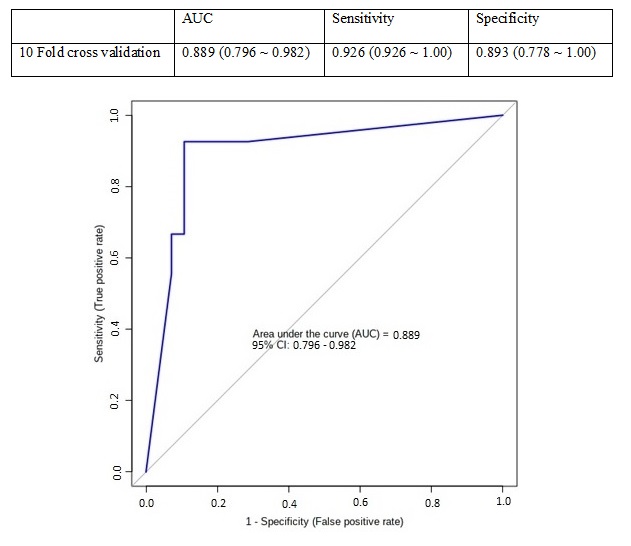


**Legend for Supplementary Figure 4**

ROC curve with 10 fold cross validation


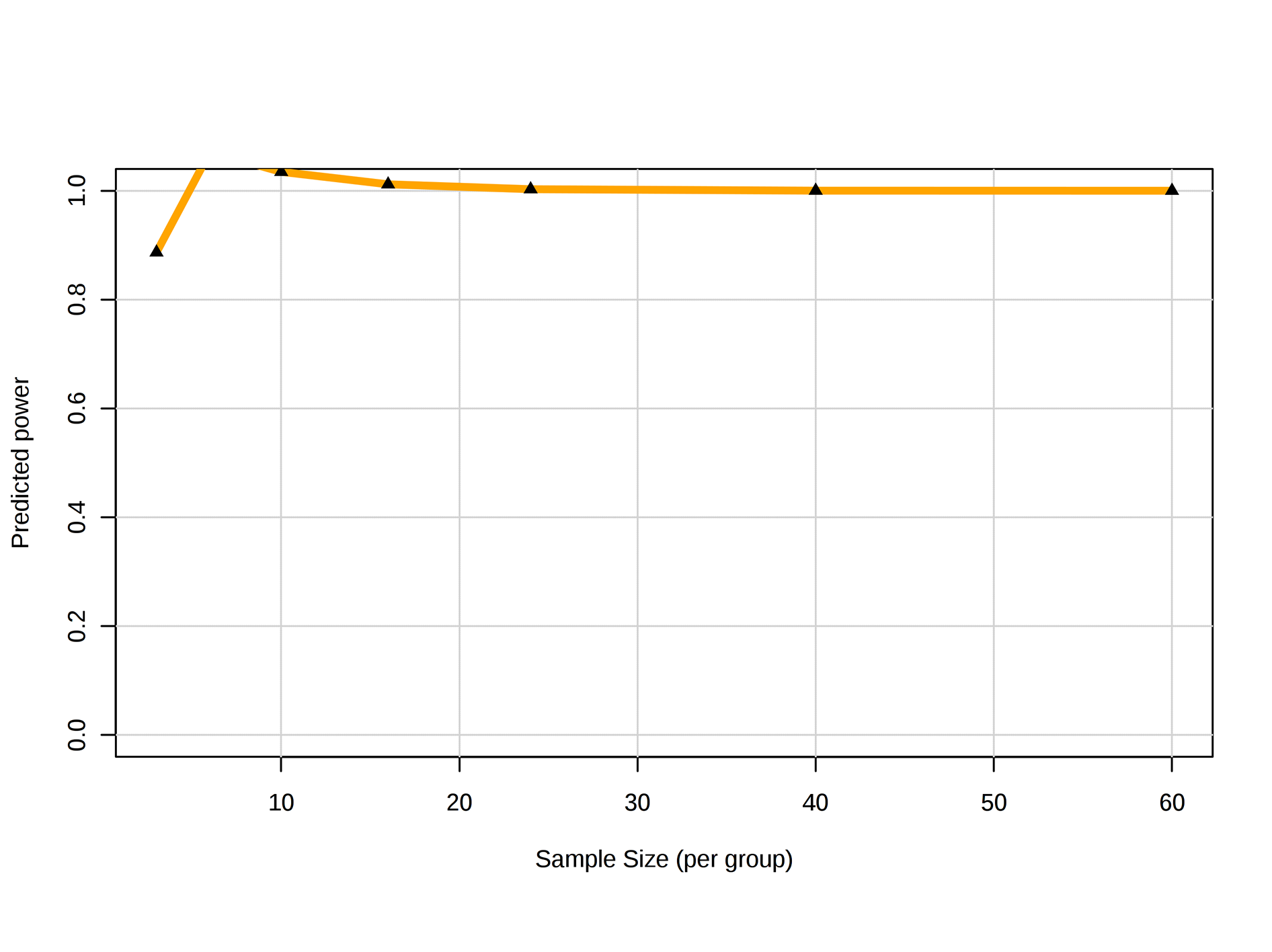


**Supplementary Figure 5:**

Sample size estimation analysis depicting the number of animals required for the study. The x-axis represents the predicted power and the y-axis represents the sample size (per group).
